# Nonatheromatous Coronary Kink Causing Angiographic Obstruction: A Rare Structural Anomaly

**DOI:** 10.1155/2023/6626263

**Published:** 2023-08-21

**Authors:** Nikita Jhawar, Abhiram Prasad, S. Michael Gharacholou

**Affiliations:** ^1^Department of Internal Medicine, Mayo Clinic, Jacksonville, FL 32224, USA; ^2^Department of Cardiovascular Medicine, Mayo Clinic, Jacksonville, FL 32224, USA; ^3^Department of Cardiovascular Medicine, Mayo Clinic, Rochester, MN 55905, USA

## Abstract

Ischemic symptoms may be explained by a multitude of coronary pathologies, including coronary artery tortuosity, atherosclerosis, fibromuscular dysplasia, vasculitis, coronary vasospasm, or microvascular disease. We present an unusual case of coronary kinking in a patient presenting with exertional jaw pain in the absence of atherosclerotic risk factors. Multimodality imaging, coronary imaging, and coronary physiology helped establish the diagnosis and guide management.

## 1. Case Presentation

**Figure 1:**
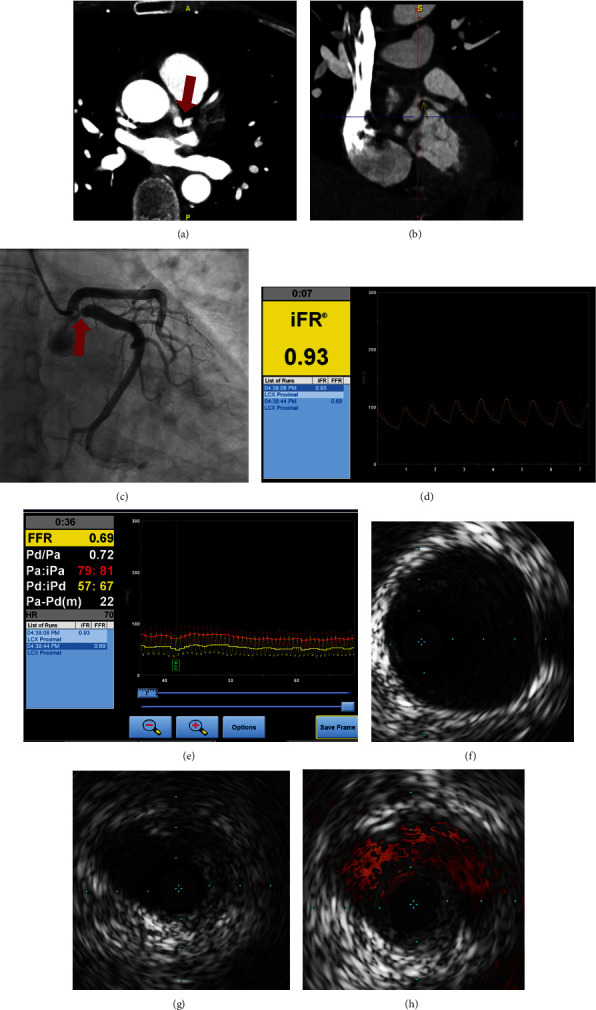
(a and b) Coronary CT angiography demonstrating proximal left circumflex artery blockage. (c) Coronary angiography showing 70% stenosis of the left circumflex artery. (d and e) Discrepant iFR and FFR results. (f) Intravascular ultrasound showing normal reference segment distal to the proximal lesion. (g) Eccentric narrow orifice without atherosclerotic plaque with a minimal lumen area of 6.7 mm^2^. (h) Color-flow image of eccentric narrow orifice without evidence of atherosclerotic plaque.

**Figure 2:**
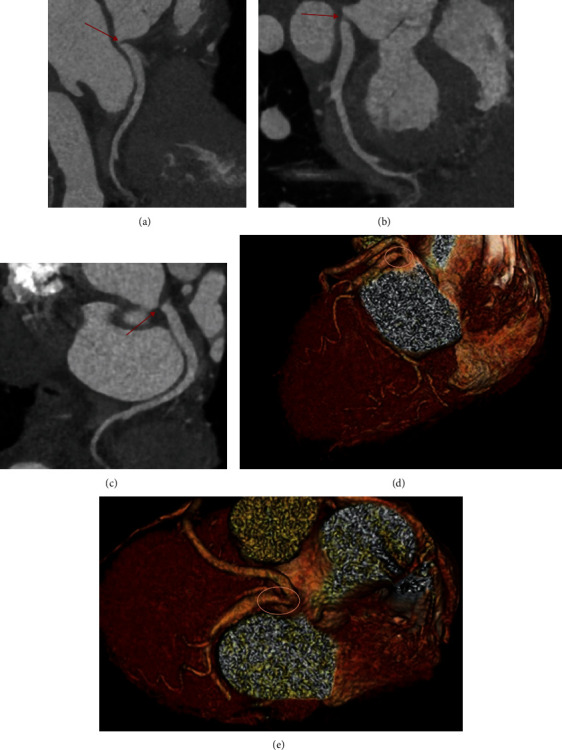
(a–c) Curved multiplanar images by coronary CT angiography noting the kink in the ostial left circumflex artery delineated by the red arrow. (d and e) CT 3D volume reconstructions of the proximal left coronary vessels and the left main artery illustrate the ostial left circumflex kink (oval) about the left main coronary bifurcation.

A healthy 56-year-old woman presented with exertional jaw discomfort relieved by rest. The patient noticed that her symptoms would begin after her heart rate exceeds 120 bpm. She had no pertinent cardiac history, traditional cardiac risk factors, or non-traditional risk factors, such as lupus, psoriasis, radiation therapy, or malignancy. The patient also denied taking naturopathic supplements and had no chronic medications. An exercise echocardiogram with standard Bruce protocol showed very good functional capacity (11.8 METS; 10:47 minutes:seconds) and demonstrated 1 mm downsloping ST segment depressions without segmental wall motion abnormalities in the anterolateral leads. She did not experience chest pain with the stress test and achieved a peak heart rate of 162 bpm and peak blood pressure of 152/92 mmHg. The patient underwent coronary computed tomography (CT) angiography demonstrating severe, isolated stenosis of the proximal left circumflex artery (Figures 1(a) and 1(b)). They were no other lesions on coronary CT, and the calcium score was 0. Coronary angiography confirmed left coronary dominance and ostial circumflex stenosis of 70% (Figure 1(c)). The lesion remained unchanged with intracoronary nitroglycerin. The instantaneous wave-free ratio (iFR) was normal (0.93), but the fractional flow reserve (FFR) obtained with intravenous adenosine was abnormal (0.69; Figures 1(d) and 1(e)). Due to these discrepant findings, intravascular ultrasound (IVUS) was performed, which did not reveal any atheromatous plaque, calcification, or intramural hematoma (Figures 1(f), 1(g), and 1(h)). Curved multiplanar images by coronary CT angiography and CT three-dimensional (3D) volume reconstructions of the proximal left coronary vessels also illustrated an ostial left circumflex artery kink (Figure 2). These collective findings suggested a fold or “kink” in the proximal vessel. The patient was treated medically with metoprolol, but due to side effects was switched to low-dose amlodipine to prevent anginal symptoms during exercise. Revascularization (percutaneous or surgical) was discussed but the patient preferred conservative management. Over two years of follow-up, the patient has remained stable without clinical events and antianginal therapy had resolved her jaw symptoms.

## 2. Discussion

We share a unique case of likely congenital coronary kinking causing stable angina for which there is a lack of consensus on optimal management. Other cases of coronary kinking have also been reported. Graft kinking can occur after coronary artery bypass surgery because of surgical technique and graft length [1]. In such cases, FFR can be used to clarify coronary stenosis, and management involves medical therapy, stenting, or surgical repair [1]. Kinking has also been seen after left internal mammary artery graft failure [2]. In this situation, vasodilators like nitroglycerin can distinguish kinks from spasms, and treatment may require stent placement to relieve the kink [2]. Coronary kinking has also been associated with aortic and mitral valve replacement surgeries due to tightening of the tissue adjacent to the coronary artery during suturing. Due to the resulting functional stenosis from suture traction, coronary perfusion can be compromised and result in ischemia or infarction [3].

We present a rare situation of congenital coronary kinking, which is understudied [4–6]. Symptomatically, this structural anomaly can mimic other arteriopathies, such as fibromuscular dysplasia, atherosclerotic plaque, or vasculitis [7]. Diagnosis is best made with multi-modality coronary imaging, such as coronary CT or IVUS, demonstrating an absence of atherosclerotic plaque or hematoma. Although discrepancies between iFR and FFR have been reported and may be present in approximately 15% of cases, the differences are often close to borderline values. We hypothesize that the marked difference between the resting and hyperemic index may relate to a healthy microcirculation that dramatically increased hyperemic flow, whereas potentially altering the geometry of the proximal kink. Discordance between iFR and FFR may also be related to the focal nature of the kink, which influences separation forces that tend to be associated with a small gradient at rest (iFR), but a large increase in the gradient with hyperemia (FFR) [8]. The FiGARO (FFR versus iFR in the Assessment of Lesions of Hemodynamic Significance, and an Explanation of Their Discrepancies) prospective study found that significantly more iFR/FFR discrepancies were associated with the right coronary artery (RCA) as opposed to the left coronary artery [9]. One explanation for this finding could be that maximum coronary flow in the RCA occurs during late systole or early diastole, but iFR is measured during mid-diastole. Other predictive variables for discrepant iFR and FFR were found to be younger age, male sex, and high estimated glomerular filtration rate [9].

Coronary kinking the ostial left circumflex artery poses a revascularization dilemma given the absence of a proximal landing zone, though an inverted provisional stent strategy could be considered. Additionally, coronary artery bypass grafting may fail due to relatively normal coronary competitive flow. Potential complications of coronary kink are poorly studied, with only anecdotal reports indicating possible progression to ischemia-induced arrhythmia, sudden coronary occlusion, and myocardial infarction [4]. Furthermore, studies are needed to investigate the role of medical versus revascularization strategies for these seemingly rare findings.

## 3. Conclusion

A coronary kink should be considered in patients with angiographic obstruction but without plaque or hematoma on IVUS and who have few cardiac risk factors. Coronary physiology may help quantify the depth of ischemia associated with the lesion. Anti-anginal medications can be used to manage symptoms, as demonstrated by our case, but more research is needed to investigate the utility of percutaneous coronary interventions versus bypass grafting in treating cases refractory to medical therapy.
